# Genome interpretation: Clinical correlation is recommended

**DOI:** 10.1016/j.atg.2015.07.002

**Published:** 2015-07-22

**Authors:** Michael M. Segal

**Affiliations:** 27 Crafts Road, Brookline, MA 02467, USA

In a brain MRI report, the following words often appear: “clinical correlation is recommended”. These words signify that inadequate clinical information was provided, or that an unexpected finding on the MRI should be assessed clinically. “Clinical correlation is recommended” is less common in a report about a single gene or simple gene panel. This is because the very act of ordering the test conveys much of what is important about the clinical situation, and only rarely is further information needed.

Genetics labs are moving into new territory as they adopt next-generation genomic sequencing. When moving beyond single gene tests and simple panels, more clinical correlation is needed. The complexity of interpretation becomes similar to a brain MRI, only more so.

In an exome, thousands of variants are found. Even after comparing to other family members, and using estimates of variant pathogenicity, many genes must be considered. Sometimes clinical correlation can be as simple as using the key clinical finding, assuming that you know which finding is key. But sometimes the situation is more complicated: variants are found in a gene that hadn't been considered clinically, or two genes are needed to explain the clinical picture, and more clinical correlation is needed.

For brain MRI scans, this clinical correlation is institutionalized as neuroradiology rounds held several times a week by many neurology inpatient services. How should the analogous clinical correlation for genomic interpretation be structured?

The core principle of clinical correlation should be that detailed information about the patient's findings, the phenotype, is combined with detailed information in the annotated variant table, the genotype. Since this is done in the wider context of considering all known phenotypes, the result is a “genome–phenome” analysis.

In what venue should the “aha” of this clinical correlation occur? Each venue has plusses and minuses:

• *External lab*: For the external lab to do the clinical correlation, the referring clinicians need to send phenotypic information to the lab, with the full detail used in clinical reasoning, including onset ages of findings and pertinent negatives (Segal et al., 2014). The lab needs solid clinical expertise to use this information, and needs to ask for and receive more clinical information as unanticipated gene abnormalities are detected. For most labs, this would represent an increase in clinical expertise and clinical communication, and for referring clinicians, this would represent a need to submit more information in a fashion that labs can process efficiently, not as long copies of records from which it is difficult or impossible to extract the relevant findings, let alone onset information or pertinent negatives.
• *Hospital-based*: For hospital-based clinicians to do the clinical correlation, the clinicians who know the patient need the expertise to work with an annotated variant table. The sequencing and annotation could be done internally, or by an external lab, which can share an annotated variant table annotated variant table under CLIA rules without triggering a “reporting event”. For most clinical geneticists, analyzing a variant table would represent an increase in expertise, requiring significant training. If sequencing were to be done internally rather than in an external lab, this would represent an increase in genomic expertise needed in the hospital, with a danger of falling behind external labs that have the scale to use crucial advanced capabilities such as family-aware variant calling and exome sequencing that includes enhanced coverage of disease genes (Segal et al., 2015).
• *Hybrid*: Genome and phenome expertise can be united by having meetings of clinical and genomic experts similar to neuroradiology rounds or tumor boards. This hybrid venue involves delays and overhead of big teams, but unites deep expertise in both genome and phenome. The efficacy of the process would depend on the quality and efficiency of this communication between clinical and genomic experts.

Each of these models involves considerable training or overhead to combine genome and phenome information. However, the ability to do so is greatly facilitated by software tools that help combine genomic and clinical expertise. Such software has developed in a stepwise process and is now in use among clinicians and lab experts:

• *Clinical diagnosis became more systematized*: Clinicians have become increasingly open to computerized assistance in clinical diagnosis. The openness came in part from younger clinicians who cope with considering thousands of diagnoses the same way that they deal with other areas of daunting information — by using computerized tools. This openness has encouraged systematized clinical terminology, detailed descriptions of findings in known diseases, and the software to combine this information (Segal and Leber, 2012).
• *Genome–phenome analysis became more systematized*: Building on clinical diagnostic software, genome–phenome analysis is becoming systematized in a way that is hypothesis-independent as to the mechanism of inheritance, the number of genes involved, and which clinical findings are important. Computational metrics such as “pertinence” can bridge the divide between genome and phenome information by highlighting genes that influence the differential diagnosis (Segal et al., 2015).
• *Communication improves as its power is demonstrated*: When clinicians are part of the process of genome interpretation, they see the importance of providing robust phenotypic information and helping curate information about known phenotypes. Seeing the difference between “garbage in, garbage out” and “quality in, quality out” is worth a thousand exhortations.

As labs go through the cultural shift from single gene tests to genomic analysis, it is crucial to focus on the process of clinical correlation, the venue in which it is done, and the tools that can help get clinical and genomic experts “on the same page”.

## Funding source

Some of the work on which this commentary is based has been supported by NIH grants and contracts to the author (National Library of Medicine HHSN276201000026C and 5R44LM011585, and National Human Genome Research Institute 1R43HG006974 and 2R42HG006974).

## Conflict of interest

The author is employed by SimulConsult, which produces genome analysis software.
